# A Novel Loop-Mediated Isothermal Amplification Assay for Serogroup Identification of *Neisseria meningitidis* in Cerebrospinal Fluid

**DOI:** 10.3389/fmicb.2015.01548

**Published:** 2016-01-12

**Authors:** DoKyung Lee, Eun Jin Kim, Paul E. Kilgore, Hideyuki Takahashi, Makoto Ohnishi, Jun Tomono, Shigehiko Miyamoto, Daisuke Omagari, Dong Wook Kim, Mitsuko Seki

**Affiliations:** ^1^Department of Pharmacy, College of Pharmacy, Hanyang UniversityAnsan, South Korea; ^2^Institute of Pharmacological Research, Hanyang UniversityAnsan, South Korea; ^3^Department of Pharmacy Practice, Eugene Applebaum College of Pharmacy & Health Sciences, Wayne State UniversityDetroit, MI, USA; ^4^Department of Bacteriology I, National Institute of Infectious DiseasesTokyo, Japan; ^5^Kaneka, Co., Ltd., OsakaJapan; ^6^Nihon University School of DentistryTokyo, Japan; ^7^Dental Research Center, Nihon University School of DentistryTokyo, Japan

**Keywords:** *Neisseria meningitidis*, serogroup identification, loop-mediated isothermal amplification, meningitis, cerebrospinal fluid, children

## Abstract

We have developed a novel *Neisseria meningitidis* serogroup-specific loop-mediated isothermal amplification (LAMP) assay for six of the most common meningococcal serogroups (A, B, C, W, X, and Y). The assay was evaluated using a set of 31 meningococcal LAMP assay positive cerebrospinal fluid (CSF) specimens from 1574 children with suspected meningitis identified in prospective surveillance between 1998 and 2002 in Vietnam, China, and Korea. Primer specificity was validated using 15 *N. meningitidis* strains (including serogroups A, B, C, E, W, X, Y, and Z) and 19 non-*N. meningitidis* species. The *N. meningitidis* serogroup LAMP detected down to ten copies and 100 colony-forming units per reaction. Twenty-nine CSF had *N. meningitidis* serogroup identified by LAMP compared with two CSF in which *N. meningitidis* serogroup was identified by culture and multi-locus sequence typing. This is the first report of a serogroup-specific identification assay for *N. meningitidis* using the LAMP method. Our results suggest that this assay will be a rapid, sensitive, and uniquely serogroup-specific assay with potential for application in clinical laboratories and public health surveillance systems.

## Introduction

*Neisseria meningitidis* is a gram-negative β-proteobacterium and member of the bacterial family Neisseriaceae (Stephens et al., 2007). The meningococcal polysaccharide capsule and outer membrane proteins have been identified as *N. meningitidis* virulence factors (Stephens, 2009). *N. meningitidis* is principally subdivided into 12 serogroups based on the capsular polysaccharide type (A, B, C, E, H, I, K, L, W, X, Y, and Z; Harrison et al., 2013). Globally, the majority of invasive meningococcal disease has been attributable to six serogroups designated A, B, C, W, X, and Y (Stephens et al., 2007; Stephens, 2009; Verma and Khanna, 2012).

The geographic distribution and epidemic potential of *N. meningitidis* may vary by serogroup. In Europe and other industrialized countries, serogroups B and C are major causes of invasive meningococcal disease (Harrison et al., 2009). The majority of meningococcal disease in European countries, which ranges in incidence from 0.2 to 14 cases per 100,000 (Harrison et al., 2009), is caused by serogroup B strains, particularly in countries that have introduced meningococcal serogroup C conjugate vaccines. More recent reports have noted increases in serogroup W and Y meningococcal disease (Campbell et al., 2009; Ladhani et al., 2012). In the Americas, the majority of meningococcal disease is caused by serogroups B and C (Stephens et al., 2007). The emergence of serogroup W and Y has been noted in some countries where surveillance is present (Chiavetta et al., 2007). In Africa, *N. meningitidis* is the leading cause of severe, life-threatening meningitis and has been responsible for thousands of cases and scores of deaths across sub-Saharan meningitis belt countries (Alonso et al., 2006). Seasonal large-scale epidemics caused by *N. meningitidis* serogroup A have been frequent in Sub-Saharan Africa ranging from Senegal to Ethiopia (WHO, 2011). The introduction of meningococcal serogroup A conjugate vaccines in countries of Sub-Saharan Africa has led to significant reductions in serogroup A epidemics (Kristiansen et al., 2014). Serogroup C, serogroup X and, most recently, serogroup W disease have also been reported (Harrison et al., 2009). The emergence of serogroup W meningococcal disease in Africa was associated with a large epidemic among Hajj pilgrims in 2000 and a large-scale meningitis outbreak in Burkina Faso during 2002 (Alonso et al., 2006). In Asia, serogroup B has been identified as the leading cause of meningococcal disease in Thailand and Taiwan (Harrison et al., 2009). *N. meningitidis* serogroups A, C, X, and Y have been identified in these and other Asian countries (Stephens et al., 2007; Harrison et al., 2009; Kim et al., 2012b).

Traditionally, invasive disease due to *N. meningitidis* has been diagnosed using specialized bacterial culture media and reagents (e.g., anti-sera) used for identifying *N. meningitidis* serogroups (Corless et al., 2001). While traditional gram staining and observation using light microscopy are used for *N. meningitis* detection, these techniques do not permit serogroup identification (Chanteau et al., 2006). The diagnosis of *N. meningitidis* invasive disease by culture testing requires a well-equipped laboratory with appropriate biosafety equipment and procedures (Borrow et al., 2014). In some settings, rapid immunochromatographic antigen detection testing has been applied for detection of *N. meningitidis* but these assays are limited by relatively low diagnostic sensitivity (Chanteau et al., 2006). Traditional diagnostic assays also have limited utility in many populations and health systems where inappropriate use of antibiotics continues despite global increases in antibiotic resistance (Levy and Marshall, 2004). In such settings, detection of *N. meningitidis* may be problematic due to inhibition of bacterial growth due to antibiotics present in human clinical samples (Laxminarayan et al., 2013). In such cases, isolation of bacteria from patients’ cerebrospinal fluid (CSF) is time-consuming and may yield little or no growth.

Given the geographic and temporal variations in meningococcal strains causing both epidemic and sporadic disease, the serogroup identification of *N. meningitidis* to identify circulating serogroups is important as a part of disease control and surveillance programs. Efforts to improve diagnostics for *N. meningitidis* have included development of a rapid immunochromatographic assay that uses monoclonal antibodies against polysaccharides for identification of serogroups A, W, C, and Y. Use of this assay has been most prominent in endemic regions of Africa (Chanteau et al., 2006). A number of PCR-based methods have been developed to identify major *N. meningitidis* serogroups by targeting the polysialyltransferase *siaD* gene or other genes involved in serogroup-specific capsule synthesis (Taha et al., 2005; Fraisier et al., 2009). In addition, real-time and multiplex PCR assays for serogroup identification of *N. meningitidis* have been developed (Fraisier et al., 2009; Zhu et al., 2012; Doyle and Jennison, 2013). However, equipment required for conventional and real-time PCR assays is relatively expensive, and PCR methods are complex to perform in resource-limited laboratory settings found in many developing countries where *N. meningitidis* infections have been reported.

The loop-mediated isothermal amplification (LAMP) method (Notomi et al., 2000, 2015) offers an attractive alternative to bacterial cultivation and PCR. The LAMP assay uses four different primers to identify six distinct regions on the target gene, resulting in a greater specificity than conventional PCR. Additional primers (i.e., the loop primers designated LF and LB) designed to anneal the loop structure in LAMP can be used to increase sensitivity and specificity of the LAMP reaction (Tomita et al., 2008). During the LAMP reaction, a small amount of DNA can be detected due to the high amplification capacity of the assay. In addition, the LAMP assay does not require the use of complicated procedures, equipment, or machines. For these reasons, the LAMP assay is considered more rapid, efficient, simpler, and more economical than other diagnostic methods.

Recently, we developed a LAMP-based assay (Lee et al., 2015) that demonstrated high sensitivity and specificity for *N. meningitidis* detection in CSF. In the present study, we report the development of a LAMP-based *N. meningitidis* serogroup identification assay and the evaluation of this assay’s ability to detect specific *N. meningitidis* serogroups in CSF. This is the first report of *N. meningitidis* serogroup-specific identification using the LAMP method.

## Materials and Methods

### Bacterial Strains

This study used 35 standard reference strains, including 15 *N. meningitidis*; serogroups A (HY0001 and NIID1), B (HY0002, H44/76, and NIID2), C (HY0003 and NIID3), E (NIID8), W (HY0006 and NIID93), X (HY0004 and NIID4), Y (HY0005 and NIID5), and Z (NIID6): nine non-meningococcal *Neisseria* species; *N. gonorrhoeae* NIID9, *N. flavescens* NIID10, *N. denitrificans* NIID11, *N. elongata* NIID12, *N. canis* NIID13, *N. cinerea* NIID14, *N. lactamica* NIID85, *N. mucosa* NIID16, and *N. sicca* NIID17: and 10 other bacterial strains; *Streptococcus pneumoniae* ATCC 49619, *Staphylococcus aureus* ATCC 29212, *Klebsiella pneumoniae* ATCC 700603, *K. oxytoca* ATCC 700324, *Pseudomonas aeruginosa* ATCC 27853, *Escherichia coli* ATCC 25922, *Enterococcus faecalis* ATCC 700324, *Mycobacterium tuberculosis* ATCC 27294, and *Haemophilus influenzae* ATCC 9007 and IID984 (Lee et al., 2015; Supplemental Materials, **Table 1**).

**Table 1:** Loop-mediated isothermal amplification (LAMP) primer sets for serogroup identification of *N. meningitidis.*

Serogroup primer name	Primer sequence	Length (base pairs)
Serogroup A	Sequence 5′–3′; Reaction temperature, 65°C	

MenA_F3	CGT AAA TGA AAT TTG GAC AG	20
MenA_B3	TTA TGA TCT TCT TCA TAG GGT A	22
MenA_FIP	GAA CTC TAA TCT GAA CCA AAA TTG AGA GTT GAC ATG AAA CTC AGC ACA G	49
MenA_BIP	CCT ACA GCT AAC AGA TAT TCT AGA AAA CGA ATA GTT TCG TAT GCC TTC	48
MenA_LF	ATA GAT GAA CTT AAA GTT CT	20
MenA_LB	GGA AGC ACT CTA TTA AAA ATA ATC	24

Serogroup B	Sequence 5′–3′; Reaction temperature, 65°C	

MenB_F3	AAA CCC TCG GCT GGT AG	17
MenB_B3	CTT AAT AAT CTC TAA GTG TTC TTG	24
MenB_FIP	GGC CAG GCC TAT AAT TCC TTC CTT TTC TAA TTG AGC CCC T	40
MenB_BIP	CAC CCT CAA CCC AAT GTC TTT CTC ATT TCA GTG TTT TCC ACC	42
MenB_LB	GGA GAG TTA ATT ATT AAC TTA ATT CAA	27

Serogroup C	Sequence 5′–3′; Reaction temperature, 65°C	

MenC_F3	TGC TCT TCA ATT AAA GCG G	19
MenC_B3	GGT AAC AAT TAA TCC CCG TCT	21
MenC_FIP	CCT ACT ACC CAA TGT CTG TCA ATT TTG TTG GGC TGT ATG GTG	42
MenC_BIP	AGT CGA TGT CAG TCC AAT AAT TCC TGT AGT GAT TAA TGA ACC CCC T	46
MenC_LF	GGG CAA ATC GTG ATT G	16
MenC_LB	GGG TTG TTA AAT AAA TTA GTG G	22

Serogroup W	Sequence 5′–3′; Reaction temperature, 64°C	

MenW_F3	GAC AAT AAG TTA CAA AAC CGT ATC	24
MenW_B3	TCA CCA GTT TTA AAA ACA CAA CC	23
MenW_FIP	CTC ACT TTC TGA TGT CAT GAT CAG GTT ATT CAA AGG TGA ATC TTC CGA	48
MenW_BIP	GGA AGG CAT GGT GTA TGA TAT TCC GTT ACT GTA ATC ATT CGC TCC	45
MenW_LF	TCT GTA TTT TCA TAA ATT TCC TGC	24

Serogroup X	Sequence 5′–3′; Reaction temperature, 63°C	

MenX_F3	GCC TTA TAC AAA GAC TGC G	19
MenX_B3	AAT AGG GGA TAG ATA ATT AGA GGT	24
MenX_FIP	GCC GAG TGC TAA GAA AGT AGA ATC TCA ATC AAT TCC ACT TCA GGG A	46
MenX_BIP	CCT GTT GTT GGC AAA GAA CTA CAA AAT GCA AAT TCA ATT GGT TGG	45
MenX_LF	GTC AGG TAT CTT CTG AAA CTC AAA	24
MenX_LB	ACC ATT GTA GCG GTC ATA AGT	21

Serogroup Y	Sequence 5′–3′; Reaction temperature, 63°C	

MenY_F3	TGT CAA AAC CTC CAG C	16
MenY_B3	CGC TAA ACG ATA CAT TTC CA	20
MenY_FIP	C**G**^a^G ***G***^b^ TT TGA AGA ATT GTT GAT GGT GAC A TT CCA GAA AAT GTT AG	44
MenY_BIP	**C**^a^A***C***^c^ TGC CCA CTA TAA GCA TGT TTT GAG TTG AAG AGG ATG AGT GA	44
MenY_LF	GAA TAA AAA GGA ATA TTT CGG C	22
MenY_LB	TCT TTA TTA TCT GAA GAA GAT AGC	24

*^a^Specific sequence of *N. meningitidis* serogroup *Y;* ARMS: ^b^original sequence was C; ^c^original sequence was G*.

For the detection limit study; we used six *N. meningitidis* strains; serogroups A (HY0001), B (HY0002), C (HY0003), W (HY0006), X (HY0004), and Y (HY0005). Bacterial colony forming unit (CFU) was determined as follows: bacteria were inoculated on chocolate agar culture plate (Becton, Dickinson & Co., Franklin Lakes, NJ, USA) and further incubated in 5% CO_2_ at 37°C for 24 h. The bacteria were collected in 1 ml PBS and CFUs were determined by serial dilutions of culture suspensions inoculated on chocolate agar plate.

### Preparation of Chromosomal DNA

Genomic DNA was extracted from the 35 standard reference strains by the phenol-chloroform method (Lee et al., 2015). For detection limit analysis, genomic DNAs from *N. meningitidis* serogroups A (HY0001), B (HY0002), C (HY0003), W (HY0006), X (HY0004), and Y (HY0005) were used, and the concentration was determined using a NanoDrop 1000 (Thermo Fisher Scientific, Inc., Waltham, MA, USA). Approximately 2.5 fg/2 μl of a DNA template was taken as one copy of the *N. meningitidis* genome per reaction, based on the genome of *N. meningitidis* serogroup B strain MC58 [accession number AE002098; 2272351 bp (Tettelin et al., 2000)]. To ascertain the detection limit of the LAMP assay, serial 10-fold dilutions of the genomic DNA were tested in each LAMP reaction, and the results were compared with those of each culture test as described previously (Kim et al., 2012b).

For the detection limit study, triplicate LAMP testing was performed using 10-fold dilutions of genomic DNA. Two technicians tested the same samples independently to confirm the reproducibility of LAMP results as described previously (Kim et al., 2012a; Lee et al., 2015).

### LAMP Primer Design

Using PrimerExplorer V4 software^1^, we designed six serogroup identification LAMP primer sets (**Table 1**) targeting the genome sequence of each serogroup-specific gene identified (Taha et al., 2005; Fraisier et al., 2009): *sacB* (GenBank accession number FR774048) for serogroup A, *siaD* (GenBank accession number CP002424) for serogroup B, *siaD* (GenBank accession number AM421808) for serogroup C, *synG* (GenBank accession number AY234197) for serogroup W, *ctrA* (GenBank accession number AY289931) for serogroup X, and *synF* (GenBank accession number AY234201)for serogroup Y.

Due to a high level of sequence identity between *synF* and *synG* genes (Zhu et al., 2012; Doyle and Jennison, 2013), we used the LAMP method along with an amplification refractory mutation system (ARMS; Newton et al., 1989; Ikeda et al., 2007) to detect the specific sequences of *synF* (**Table 1**). The ARMS uses uniquely designed primers that enable detection of mutations (Newton et al., 1989). As a target of the present investigation, we chose two specific sequences of the serogroup Y *synF* gene. Based on the principle of ARMS, one of the two specific sequences was designed in the 5′ end of the BIP primer, and the other was designed in the second sequence of the 5′ end of the FIP primer (**Table 1**). We then added one mutation in the third sequence of the 5′ end of the BIP primer (from G to C), and an additional mutation in the fourth sequence of the 5′ end of the FIP primer (from C to G).

### LAMP and PCR Reactions

The LAMP reaction was performed with 25 μl of a mixture containing 1.6 μM each of primers FIP and BIP, 0.2 μM of primers F3 and B3, 0.4 μM of primers LF and LB, 8 U of the *Bst* DNA polymerase large fragment (New England Biolabs, Ipswich, MA, USA), 1.4 mM each of the four deoxynucleoside triphosphates, 0.8 M betaine, 20 mM Tris-HCl (pH 8.8), 10 mM KCl, 10 mM (NH4)_2_SO_4_, 8 mM MgSO_4_, 0.1% Tween 20, and 2 μl of template. The high-performance liquid chromatography-purified primers were dissolved in Tris-EDTA buffer. The mixture was incubated at 63–65°C (**Table 1**) for 60 min and then heated at 80°C for 2 min to terminate the reaction. For the detection limit study, PCR was performed as described previously (Fraisier et al., 2009).

### Analysis of LAMP Products

**FIGURE 1:**
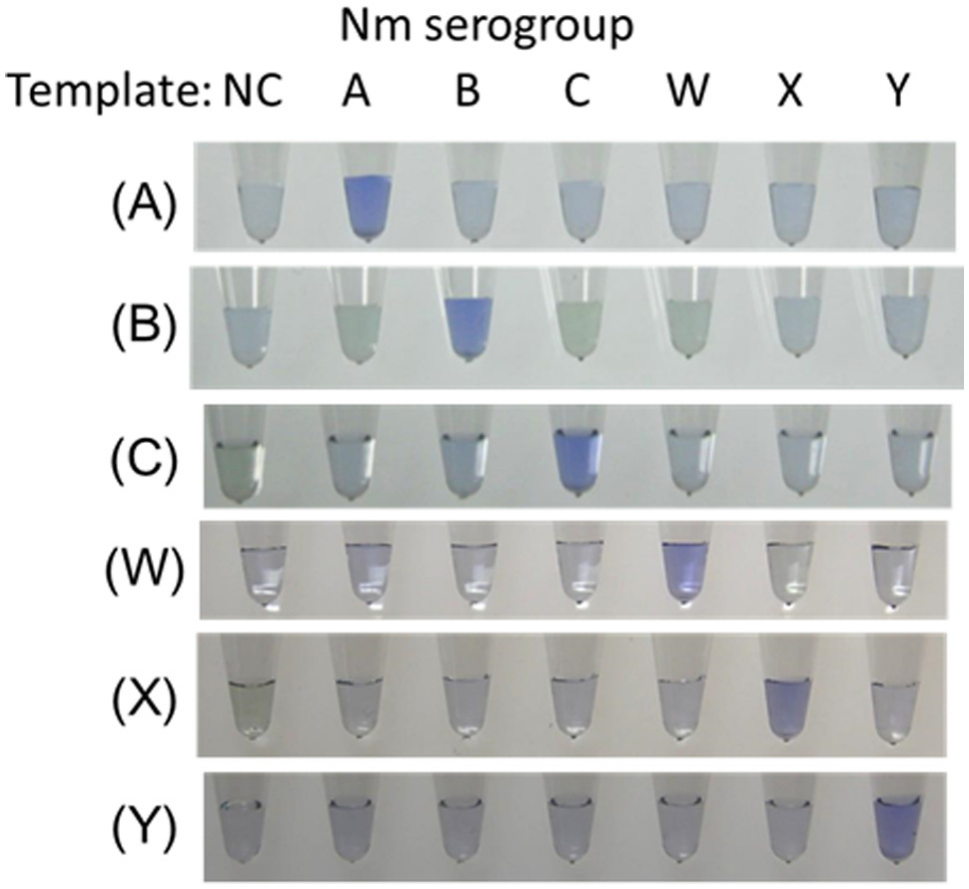
**Visual inspection of dye-mediated monitoring of the *N. meningitidis* serogroup-specific loop-mediated isothermal amplification (LAMP) assay**. The original colorless appearance of the visual inspection dye (Kaneka, Co., Ltd., Osaka, Japan) changed to blue if the reaction was positive; if reaction was negative, the dye remained colorless. (A), Results of *N. meningitidis* serogroup A-LAMP assay. (B), Results of *N. meningitidis* serogroup B-LAMP assay. (C), Results of *N. meningitidis* serogroup C-LAMP assay. (W), Results of *N. meningitidis* serogroup W-LAMP assay. (X), Results of *N. meningitidis* serogroup X-LAMP assay. (Y), Results of *N. meningitidis* serogroup Y-LAMP assay. NC, negative control.

*Neisseria meningitidis* serogroup detection was observed by the visual inspection based on its generation of turbidity proportional to the amount of amplified DNA (Mori et al., 2004; Lee et al., 2015). A Loopamp real-time turbidimeter (LA-200 real-time turbidimeter; Eiken Chemical, Co., Ltd., Tokyo, Japan) was used to monitor the turbidity in the reaction tube in real-time by reading the OD_650_ every 6 s. According to the manufacturer’s protocol (Mori et al., 2004), we used the application software for the turbidimeter to obtain the amplification time required to exceed a turbidity level of 0.1 (*Tt*). For the detection limit and specificity study, we also used electrophoretic analysis and a colorimetric visual inspection dye, Leuco Crystal Violet (LCV; D-Quick; Kaneka, Co., Ltd., Osaka, Japan; Miyamoto et al., 2015). LCV was dried down in the caps of the reaction tubes. After reactions were completed, the LAMP amplicons were mixed by inverting the tubes, and changes in color were observed to obtain test results (negative, remained colorless; positive, color change to blue: **Figure 1**).

To verify the structure of LAMP amplified products, the amplified LAMP products were sequenced using a BigDye Terminator v. 3.1 cycle sequencing kit (Applied Biosystems, Foster City, CA, USA) and an ABI PRISM 377 DNA sequencer (Applied Biosystems) according to the manufacturer’s instructions (Kim et al., 2012a; Lee et al., 2015). The target region was between F2 and B2, and the primer sequences were from the F2 and B2 regions.

### Clinical CSF Specimens

Children with suspected meningitis who were less than 5 years of age were prospectively enrolled at the participating hospitals between 1998 and 2002 (Kim et al., 2012b). CSF was streaked on commercial blood agar culture medium (Becton, Dickinson & Co., Franklin Lakes, NJ, USA), incubated in 5% CO_2_ at 37°C for 3 days, and checked daily for bacterial growth (Kim et al., 2012b). Isolates were identified using standard microbiological criteria (Isenberg, 1998). The established multi-locus sequence typing (MLST) scheme^2^ for *Neisseria* sp. was used for further genotype identification.

As described previously (Kim et al., 2012a; Lee et al., 2015), to extract bacterial DNA, CSF specimens were heated at 95°C for 3 min and centrifuged at 13,000 × *g* for 5 min. The samples were stored at -80°C. Two microliters of the supernatant were used for LAMP assays. In the previous study (Lee et al., 2015), we had tested 1,574 randomly selected CSF specimens in Korea (*n* = 470), China (*n* = 536), and Vietnam (*n* = 568) using the Nm-LAMP assay targeting specific sequences of *N. meningitidis ctrA* gene. In this study, we evaluated the serogroup identification LAMP assay using the set of 31 Nm-LAMP positive CSF specimens.

### Ethics Statement

We utilized CSF specimens preserved from our previous surveillance study (Kennedy et al., 2007). All CSF specimens utilized in this study were de-identified prior to laboratory processing and analysis. Ethical approvals for patient specimen collection during surveillance were obtained from the following ethics review committees: the Institutional Review Board of International Vaccine Institute, Seoul, Korea; the Institutional Review Board at National Institute of Hygiene and Epidemiology, Hanoi, Vietnam; and the Guangxi Zhuang Autonomous Region Center for Disease Control, Nanning, China. Each institution participated in prospective, population-based surveillance for childhood meningitis from 1999 to 2002 (Kim et al., 2004; Anh et al., 2006). During those surveillance studies, written consent was not obtained as the CSF collection was considered routine standard care for hospitalized children with suspected bacterial meningitis. For this reason, verbal consent of the parent or legal guardian present with the child during the period of hospitalization was recorded in the patient’s medical chart at the time of the clinical lumbar puncture procedure. This consent procedure was approved by these local scientific ethics review committees of the participating institutions.

## Results

### Specificity of the Serogroup Identification LAMP Assay

To evaluate the species specificity of the *N. meningitidis* LAMP primer set, we tested 35 reference strains, as described above. When approximately 1-ng whole-cell DNA (10^6^ copies of genomic DNA of each strain) was used in the LAMP reaction, amplification products from each of the related serogroup strains were observed within 30 min, whereas none of the other strains resulted in amplification products even after 60 min of incubation (**Figure 1**). We confirmed that the amplification products corresponded to the selected sequence target by sequencing using primers F2 and B2. The LAMP reaction is highly specific for each serogroup.

### Detection Limits of the Serogroup Identification LAMP and PCR Assays

The detection limits for the LAMP assays were 10 or 100 genome copies and 100 to 1,000 CFUs (25 μL of reaction mix, triplicate trials) as described in **Table 2**. The detection limits of the PCR assays were 10^3^ to 10^4^ genome copies per reaction (**Table 2**). Throughout the study, the evaluation of the LAMP reactions showed complete agreement between a white precipitate recorded by visual inspection, the real-time turbidimeter, electrophoretic analysis, and LCV. Using LCV, the colorless dye changed to blue color indicating a positive reaction. These results were evaluable under natural light without the need for UV light (**Figure 1**).

**Table 2:** Detection limits of the *N. meningitidis* serogroup-specific LAMP assay.

	Detection limit of each *N. meningitidis* serogroup					
Assay	A	B	C	W	X	Y
Each serogroup-specific LAMP	10^2^ copies^a^	10^2^	10^2^	10	10^2^	10^2^
	10^2^ cfu^b^	10^2^	10^3^	10^2^	10^2^	10^2^
Each serogroup-specific PCR^c^	10^3^ copies^a^	10^4^	10^4^	10^3^	10^3^	10^4^

*^a^Amount of DNA per reaction*.

*^b^Amount of colony forming units per reaction*.

*^c^Conventional PCR (Fraisier et al., 2009)*.

### LAMP Applied to Analysis of Clinical CSF Specimens

A group of 31 known Nm-LAMP-positive specimens (Lee et al., 2015) were also analyzed by *N. meningitidis* serogroup-specific LAMP tests. The LAMP assay identified *N. meningitidis* serogroup for 29 of the 31 *N. meningitidis*-positive CSF specimens (**Table 3**). Using a novel LAMP assay based on the ARMS principle, we succeeded in differentiating *N. meningitidis* serogroup Y from *N. meningitidis* serogroup W (**Figure 1**). Five serogroup A (5/31, 16.1%), five serogroup B (5/31, 16.1%), three serogroup C (3/31, 9.7%), five serogroup X (5/31, 16.1%), five serogroup Y (5/31, 16.1%), and six serogroup W (6/31, 19.4%) specimens were identified. Two samples were non-typable for *N. meningitidis* (2/31, 6.5%). Each serogroup-specific LAMP-amplified product was confirmed by sequencing, and the sequence results were identical to the reference sequences.

**Table 3:** Results of the serogroup-specific LAMP assay of 31 CSF samples of *N. meningitidis ctrA* positive.

	*N. meningitidis* serogroup identified by LAMP							
Country	A	B	C	W	X	Y	Non-type	Total
Vietnam	4^a^	4^b^	2	4	2	3	1	20
China	0	1	1	0	1	2	0	5
Korea	1	0	0	2	2	0	1	6
Total	5	5	3	6	5	5	2	31

*^a^Number of positive results*.

*^b^Two strains were also found to be serogroup B contained MLST ST1576*.

In the previous study (Kennedy et al., 2007), three *N. meningitidis* culture-positive (3/31, 9.7%) were confirmed and two of the three specimens were serogroup B positive using MLST (**Table 3**). The MLST scheme and database of *Neisseria* sp. are well-established and publically available at http://pubmlst.org/neisseria/. The MLST scheme and methods previously described were applied to the *N. meningitidis* isolates. Seven MLST loci were amplified by using PCR primers and the DNA sequences of each locus were compared to the same locus from the database. Two serogroup B *N. meningitidis* isolates contained an allele type 140, 5, 9, 173, 175, 34, 165 (in the order *abcZ, adk, aroE, fumC, gdh, pdhC*, and *pgm*), which has been designated as sequence type 1576 (ST1576).

Compared to the culture method, the sensitivity and specificity of the *N. meningitidis* serogroup B LAMP assay were 100% (2/2) and 89.7% (26/29).

## Discussion

The novel *N. meningitidis* serogroup-specific LAMP assay reported here demonstrated high detection rates as well as high test specificity and sensitivity. These higher detection rates found with the LAMP assay compared with conventional PCR is consistent with previous studies of LAMP assays (Kim et al., 2011, 2012a). The robust performance of the LAMP assay is made possible by the use of four different primers to identify six distinct regions on the target gene. The design of our primers for the assay described here are unique and enable clear differentiation between six major serogroups of *N. meningitidis* responsible for invasive meningococcal disease (including meningitis) found around the world.

We successfully differentiated *N. meningitidis* serogroup Y from *N. meningitidis* serogroup W using the ARMS principle (Newton et al., 1989). The first application of ARMS in LAMP primer design to detect a point mutation of target sequences was reported by Ikeda et al. (2007). The investigator added one mutation at the 5′ end of BIP primer that permitted successful detection of the point mutation. In our experiments, optimizing the design of the LAMP primer set for serogroup Y proved to be challenging because key target gene sequences (*synF* from serogroup Y and *synG* from serogroup W) share a high degree of similarity that has been also noted by other investigators (Zhu et al., 2012; Doyle and Jennison, 2013). To successfully differentiate and accurately identify serogroup Y and W strains, we created mutations at the 5′ end of both the FIP and BIP primers.

The detection limits for the serogroup-specific LAMP assays were 10 or 100 genome copies and 100 to 1,000 CFUs, and the conventional PCR assays had much lower sensitivity (10^3^ to 10^4^ genome copies) than LAMP. The evaluation of the LAMP reactions showed complete agreement between a white precipitate recorded by visual inspection, the real-time turbidimeter, electrophoretic analysis, and LCV. The results of LCV were evaluable under natural light without the need for UV light and the color change remained visible for at least 1 week (Lee et al., 2015). The experience using LCV colorimetric visual inspection dye reported here can inform and facilitate future development and application of the LAMP assay in resource-limited settings.

We used preserved de-identified CSF specimens that were collected between 1998 and 2002 in our previous study of bacterial meningitis (Kim et al., 2012b). In the previous study (Lee et al., 2015), using 1574 randomly selected CSF specimens, we detected 31 Nm-LAMP assay positive CSF specimens including three *N. meningitidis* culture-positive (3/31, 9.7%). Two of the three specimens were confirmed as serogroup B positive using MLST.

Using the meningococcus serogroup-specific LAMP assay, 29 of the 31 known Nm-LAMP-positive specimens (Lee et al., 2015) were one of six serogroup-specific positive CSF specimens. Five serogroup A (5/31, 16.1%), five serogroup B (5/31, 16.1%), three serogroup C (3/31, 9.7%), five serogroup X (5/31, 16.1%), five serogroup Y (5/31, 16.1%), and six serogroup W (6/31, 19.4%) specimens were identified and two samples were non-typable for *N. meningitidis* (2/31, 6.5%). In these results, the observation of two non-typable specimens may be due to potential discrepancies in the sensitivity of the Nm-LAMP (10 copies; Lee et al., 2015) compared with the *N. meningitidis* serogroup-specific LAMP (10 or 100 copies) assays. Other possible explanations for the observation of non-typable strains include the degradation of DNA material in stored specimens as well as the presence of *N. meningitidis* serogroups not covered by designed LAMP assays.

In settings of many developing countries where empiric use of broad-spectrum antibiotics is common, conventional bacterial culture methods have low yield and are likely to underestimate the true burden of *N. meningitidis* serogroups. During the original prospective surveillance studies, field surveys showed that over-the-counter use of antibiotics without a prescription was common in each country (Kim et al., 2012b). Thus, to better understand the epidemiology bacterial meningitis pathogens such as *N. meningitidis*, the meningococcal serogroup-specific LAMP offers an accurate alternative diagnostic test. Our results suggest that the meningococcal serogroup LAMP may provide a useful tool in areas where public health agencies wish to measure the burden of *N. meningitidis* following introduction of meningococcal vaccines.

Based on our experience in this study, the *N. meningitidis* serogroup-specific LAMP assay was easy to set-up and required no special equipment. In addition, this LAMP assay provides high-quality test results within 2 h that make it feasible and cost-effective for use. Given the simple format for the LAMP assay, this test also has potential for performance in district-level health facilities in developing countries. To confirm the LAMP assay performance characteristics compared with bacterial culture, antigen detection and PCR, further evaluation of the *N. meningitidis* serogroup identification LAMP in prospective studies are now planned.

## Author Contributions

PK, HT, MO, DK, MS, contributed the conception of this study; SM, JT, DK, MS designed the experiments; DL, EK, SM, DK, MS, DO performed the experiments; PK, HT, MO acquired samples; DL, EK, JT, SM, DO, PK, DK, MS analyzed data; DL, EK, JT, SM, DO, PK, DK, MS interpreted data; DL, EK, PK, HT, DK, MS drafted the manuscript; and DO, MO, SM, JT approved the manuscript.

## Conflict of Interest Statement

Mitsuko Seki has received research grant funding from Kaneka, Co., Ltd. Jun Tomono and Shigehiko Miyamoto are employees of Kaneka, Co., Ltd. Paul E. Kilgore serves on meningococcal vaccine speakers bureau for Pfizer, Inc. The following authors have no conflict of interests or financial disclosures to declare: DoKyung Lee, Eun Jin Kim, Hideyuki Takahashi, Makoto Ohnishi, and DongWook Kim. These statements do not alter our adherence to the journal policies on sharing data and materials.

## Footnotes

1 https://primerexplorer.jp/lamp4.0.0/index.html

2 http://pubmlst.org/neisseria/

## ABBREVIATIONS

Nm-LAMP: meningococcal LAMP
