# Supplementary material for: A Novel Loop-Mediated Isothermal Amplification Assay for Serogroup Identification of Neisseria meningitidis in Cerebrospinal Fluid
Source: Front Microbiol. 2016 Jan 12;6:1548. doi: 10.3389/fmicb.2015.01548 (PMC4709847; doi:10.3389/fmicb.2015.01548)
Supplement: Supplementary file 1 [file Table_1.PDF]

Supplementary Materials

Table 1: Bacterial strains used in this study.

Strain	serogroup	Pedigree and Source
Neisseria meningitidis		
HY0001 [a]	A	Dr. Alonso JM, Institut Pasteur, France
NIID1 [b] (NCTC 10027)	A	National Institute of Public Health in Norway
HY0002 [a]	B	Dr. Alonso JM, Institut Pasteur, Paris, France
H44/76	B	National Institute of Public Health in Norway, the causative agent of serogroup B outbreak in 1970's in Norway
NIID2 [b] (ATCC 13090)	B	ATCC culture collection
HY0003 [a]	C	Dr. Alonso JM, Institut Pasteur, France
NIID3 [b] (ATCC 13102)	C	ATCC culture collection
NIID8 [b] (ATCC 35558)	E	ATCC culture collection
HY0006 [a]	W	Dr. Alonso JM, Institut Pasteur, France
NIID93 [b]	W	A clinical isolate from a Japanese patient
HY0004 [a]	X	Dr. Alonso JM, Institut Pasteur, France
NIID4 [b]	X	Laboratory of Hygiene of the University of Amsterdam, Netherlands
HY0005 [a]	Y	Dr. Alonso JM, Institut Pasteur, France
NIID5 [b]	Y	Laboratory of Hygiene of the University of Amsterdam, Netherlands
NIID6 [b]	Z	Laboratory of Hygiene of the University of Amsterdam, Netherlands
non-meningococcal *Neisseria* species		
N. gonorrhoeae NIID9 [b] (ATCC 49226)		ATCC culture collection
N. flavescens NIID10 [b] (ATCC 13120)		ATCC culture collection
N. denitrificans NIID11 [b] (ATCC 14686)		ATCC culture collection
N. elongate NIID12 [b] (ATCC 25295)		ATCC culture collection
N. canis NIID13 [b] (ATCC 14687)		ATCC culture collection
N. cinerea NIID14 [b] (ATCC 14685)		ATCC culture collection
N. lactamica NIID85 [b]		A clinical isolate from a Japanese healthy carrier
N. mucosa NIID16 [b]		A clinical isolate from a Japanese patient
N. sicca NIID17 [b]		A clinical isolate from a Japanese patient
other bacterial strains		
Streptococcus pneumoniae ATCC 49619		ATCC culture collection
Staphylococcus aureus ATCC 29212		ATCC culture collection
Klebsiella pneumoniae ATCC 700603		ATCC culture collection
Klebsiella oxytoca ATCC 700324		ATCC culture collection
Pseudomonas aeruginosa ATCC 27853		ATCC culture collection
Escherichia coli ATCC 25922		ATCC culture collection
Enterococcus faecalis ATCC 700324		ATCC culture collection
Mycobacterium tuberculosis ATCC 27294		ATCC culture collection
Haemophilus influenzae ATCC 9007		ATCC culture collection

Haemophilus influenzae IID984 [c] (ATCC 9334)	ATCC culture collection

[a] Culture collection of Hanyang University, Ansan, Korea

[b] Culture collection of National Institute of Infectious Diseases, Tokyo, Japan

[c] Culture collection of Institute of Medical Science, The University of Tokyo, Tokyo, Japan
